# Performance of ChatGPT on UK Standardized Admission Tests: Insights From the BMAT, TMUA, LNAT, and TSA Examinations

**DOI:** 10.2196/47737

**Published:** 2023-04-26

**Authors:** Panagiotis Giannos, Orestis Delardas

**Affiliations:** 1 Department of Life Sciences Faculty of Natural Sciences Imperial College London London United Kingdom; 2 Promotion of Emerging and Evaluative Research Society London United Kingdom

**Keywords:** standardized admissions tests, GPT, ChatGPT, medical education, medicine, law, natural language processing, BMAT, TMUA, LNAT, TSA

## Abstract

**Background:**

Large language models, such as ChatGPT by OpenAI, have demonstrated potential in various applications, including medical education. Previous studies have assessed ChatGPT’s performance in university or professional settings. However, the model’s potential in the context of standardized admission tests remains unexplored.

**Objective:**

This study evaluated ChatGPT’s performance on standardized admission tests in the United Kingdom, including the BioMedical Admissions Test (BMAT), Test of Mathematics for University Admission (TMUA), Law National Aptitude Test (LNAT), and Thinking Skills Assessment (TSA), to understand its potential as an innovative tool for education and test preparation.

**Methods:**

Recent public resources (2019-2022) were used to compile a data set of 509 questions from the BMAT, TMUA, LNAT, and TSA covering diverse topics in aptitude, scientific knowledge and applications, mathematical thinking and reasoning, critical thinking, problem-solving, reading comprehension, and logical reasoning. This evaluation assessed ChatGPT’s performance using the legacy GPT-3.5 model, focusing on multiple-choice questions for consistency. The model’s performance was analyzed based on question difficulty, the proportion of correct responses when aggregating exams from all years, and a comparison of test scores between papers of the same exam using binomial distribution and paired-sample (2-tailed) *t* tests.

**Results:**

The proportion of correct responses was significantly lower than incorrect ones in BMAT section 2 (*P*<.001) and TMUA paper 1 (*P*<.001) and paper 2 (*P*<.001). No significant differences were observed in BMAT section 1 (*P*=.2), TSA section 1 (*P*=.7), or LNAT papers 1 and 2, section A (*P*=.3). ChatGPT performed better in BMAT section 1 than section 2 (*P*=.047), with a maximum candidate ranking of 73% compared to a minimum of 1%. In the TMUA, it engaged with questions but had limited accuracy and no performance difference between papers (*P*=.6), with candidate rankings below 10%. In the LNAT, it demonstrated moderate success, especially in paper 2’s questions; however, student performance data were unavailable. TSA performance varied across years with generally moderate results and fluctuating candidate rankings. Similar trends were observed for easy to moderate difficulty questions (BMAT section 1, *P*=.3; BMAT section 2, *P*=.04; TMUA paper 1, *P*<.001; TMUA paper 2, *P*=.003; TSA section 1, *P*=.8; and LNAT papers 1 and 2, section A, *P*>.99) and hard to challenging ones (BMAT section 1, *P*=.7; BMAT section 2, *P*<.001; TMUA paper 1, *P*=.007; TMUA paper 2, *P*<.001; TSA section 1, *P*=.3; and LNAT papers 1 and 2, section A, *P*=.2).

**Conclusions:**

ChatGPT shows promise as a supplementary tool for subject areas and test formats that assess aptitude, problem-solving and critical thinking, and reading comprehension. However, its limitations in areas such as scientific and mathematical knowledge and applications highlight the need for continuous development and integration with conventional learning strategies in order to fully harness its potential.

## Introduction

Natural language processing is a rapidly evolving field that has garnered significant attention in recent years. One of the key advancements in this field is the development of large language models that are capable of generating human-like responses to user prompts [1]. ChatGPT, developed by OpenAI, is one such model; it leverages deep learning techniques to generate contextually relevant and coherent text, functioning as a general-purpose dialogic agent [2]. The model is trained on a vast corpus of text with the objective of predicting the next word in a sequence. With potential applications spanning customer service, chatbots, content creation, and language translation [3], ChatGPT has also gained traction in the realm of medical and legal education [4].

The current literature has predominantly assessed ChatGPT’s performance in medical education either at the university or professional level, such as in studies involving United States Medical Licensing Examination (USMLE) questions [5,6] or doctors’ case reports [7,8]. ChatGPT’s ability to recall and apply specific knowledge to a topic, which in theory could potentially be improved by providing the model with more specialized or updated data, is often the focus of these assessments. However, this study aimed to explore a novel aspect of ChatGPT’s performance by challenging its abilities beyond past knowledge and its application in professional settings.

We evaluated ChatGPT’s performance on questions derived from various standardized admission tests in the United Kingdom, including the BioMedical Admissions Test (BMAT), Test of Mathematics for University Admission (TMUA), Law National Aptitude Test (LNAT), and Thinking Skills Assessment (TSA) examinations. These tests play a crucial role in the selection process for competitive programs in medicine, law, and mathematics, assessing applicants’ aptitude skills to ensure they possess the necessary knowledge and abilities for their chosen field of study.

By examining ChatGPT’s performance on these tests, we aimed to understand its potential as an innovative supplemental tool for UK education and test preparation in the United Kingdom, in contexts such as small group learning or as a virtual tutor. Our analysis not only highlights the novelty of our approach, which focuses on university admission rather than professional development, but also offers insights into ChatGPT’s capabilities and limitations within specific educational contexts. We hope our results serve as a catalyst for discussions on how current education can foster the development of more effective learning tools and strategies using artificial intelligence tools like ChatGPT.

## Methods

We selected standardized UK admission tests (BMAT, TMUA, TSA, and LNAT) for our study to cover a diverse range of topics in the domains of aptitude skills, scientific knowledge and applications, mathematical thinking and reasoning, critical thinking, problem-solving, reading comprehension, and logical reasoning. This ensured a comprehensive evaluation of ChatGPT’s performance across various subject areas.

To create a data set of questions, we gathered publicly available resources and official materials. For the BMAT, TMUA, and TSA, we used past paper questions from the 3 most recent examination years (2019-2022). In contrast, for the LNAT, we relied on a past paper from 2010, as it was the only one accessible. The final data set comprised 509 questions in total, including 180 from the BMAT, 120 from the TMUA, 84 from the LNAT, and 125 from the TSA.

We used the legacy GPT-3.5 model of ChatGPT for this study. To ensure consistency in our evaluation, we exclusively used multiple-choice questions. Text-based questions were incorporated by copying and pasting the content directly, while mathematical questions without graphs and questions containing tables were formatted using LaTeX for proper structure and readability. We excluded essay-writing tasks from our analysis to mitigate potential personal bias in assessing ChatGPT’s responses, even with the availability of a mark scheme.

The assessment encompassed section 1 (Thinking Skills) and section 2 (Scientific Knowledge and Applications) of the BMAT, paper 1 (Mathematical Knowledge and Application) and paper 2 (Mathematical Reasoning) of the TMUA, section A of paper 1 and paper 2 (Comprehension and Reasoning) of the LNAT, and section 1 (Problem Solving and Critical Thinking) of the TSA. We recorded the total number of questions attempted by ChatGPT and the number of correct responses provided by the model during the evaluation process. Additionally, we estimated ChatGPT’s exam score and candidate percentage ranking based on its performance and compared it to students who took the exam.

To assess the difficulty of questions, we divided them into quartiles 1 and 2 (easy to moderate difficulty) and quartiles 3 and 4 (hard to challenging difficulty), under the assumption that difficulty increases with every question. The performance of ChatGPT based on correct responses was assessed using a binomial distribution test. Performance based on estimated test scores between sections of the same exam was evaluated using a paired-sample 2-tailed *t* test. All statistical analyses were performed with SPSS (IBM Corp), and statistical significance was set at *P*<.05.

## Results

**Figure 1:**
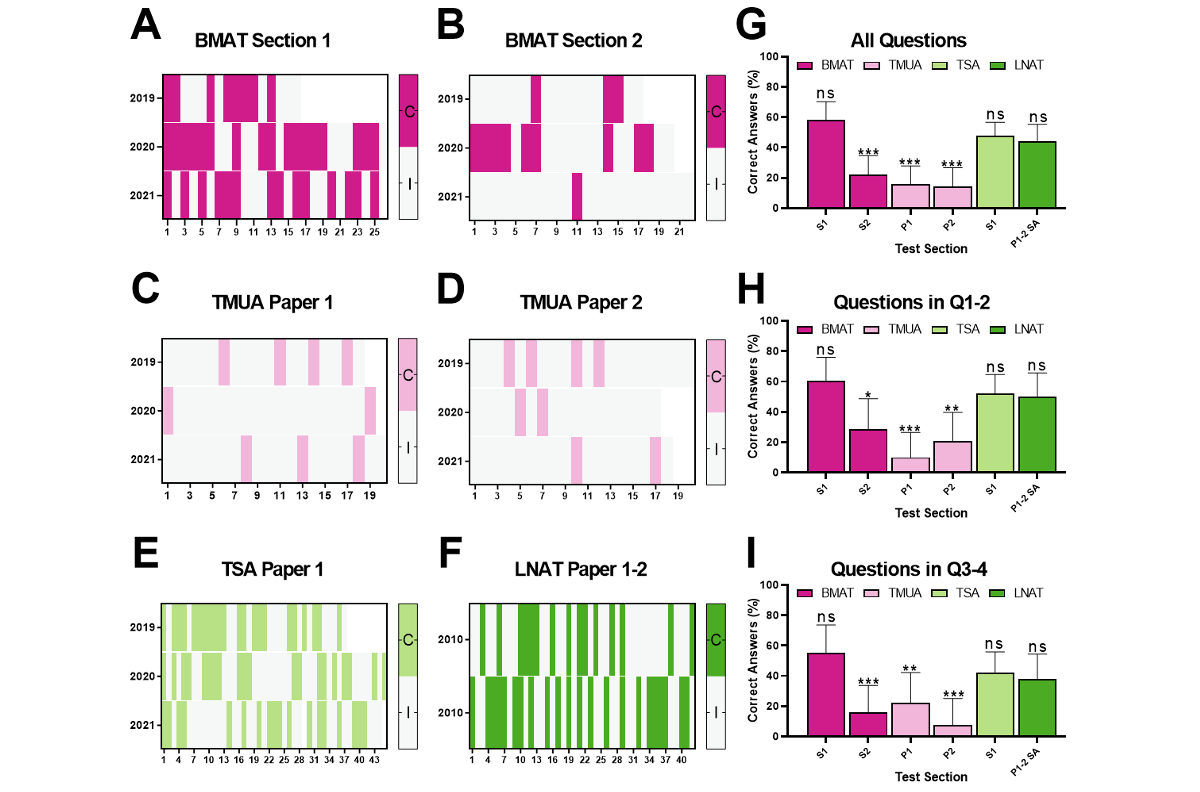
ChatGPT’s response accuracy for each question on the (A) BMAT section 1 and (B) section 2, (C) TMUA paper 1 and (D) paper 2, (E) TSA section 1 and (F) LNAT paper 1 and paper 2 admission tests, as well as the (G) overall proportion of correct responses for all questions attempted and (H) based on question difficulty for quartiles 1 and 2 and (I) quartiles 3 and 4 when considering exams from all years. BMAT: BioMedical Admissions Test; LNAT: Law National Aptitude Test; P: paper; S: section; TMUA: Test of Mathematics for University Admission; TSA: Thinking Skills Assessment; Q: quartile. ns: not significant; *P<.05,**P<.01, ***P<.001.

**Figure 2:**
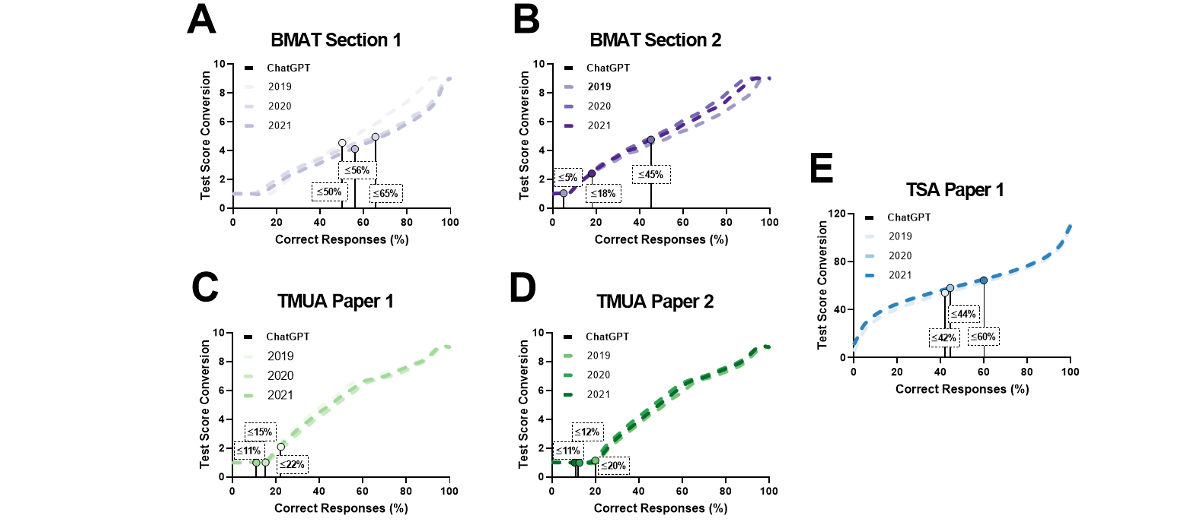
Estimated test scores derived from ChatGPT’s performance, measured as the percentage of questions answered correctly on the (A-B) BMAT, (C-D) TMUA, and (E) TSA; official performance data for the Law National Aptitude Test (LNAT) were unavailable. BMAT: BioMedical Admissions Test; TMUA: Test of Mathematics for University Admission; TSA: Thinking Skills Assessment.

**Figure 3:**
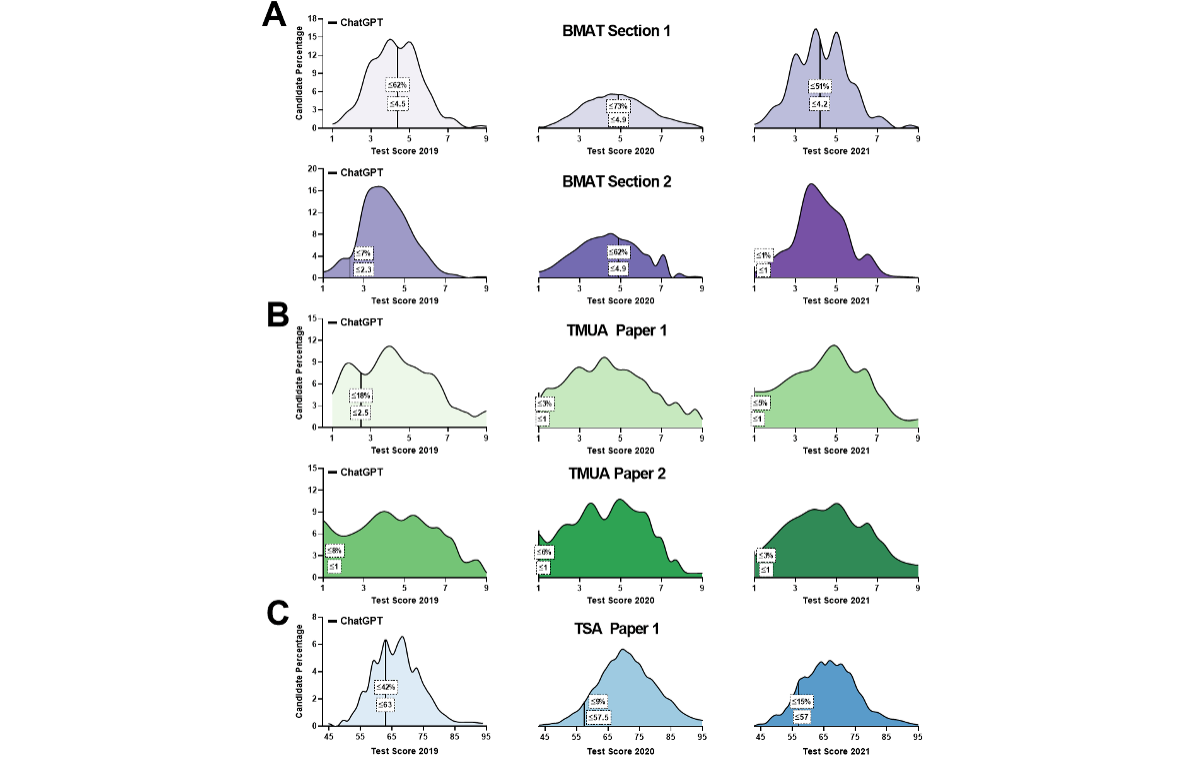
Estimated candidate percentage rankings for ChatGPT, based on its performance in terms of the percentage of questions answered correctly on the (A) BMAT, (B) TMUA, and (C) TSA, compared to students who took the exam; official performance data for the Law National Aptitude Test (LNAT) were unavailable. BMAT: BioMedical Admissions Test; TMUA: Test of Mathematics for University Admission; TSA: Thinking Skills Assessment.

ChatGPT’s performance exhibited notable variation across the different tests assessed, with some discernible patterns based on exam type and section (Table 1, Figures 1-3).

When accumulating the exams from all years, the overall proportion of correct responses was significantly different and lower than incorrect responses in BMAT section 2 (*P*<.001) and TMUA paper 1 (*P*<.001) and paper 2 (*P*<.001). No significant differences between correct and incorrect responses were seen in BMAT section 1 (*P*=.2), TSA section 1 (*P*=.7), and section A of LNAT papers 1 and 2 (*P*=.3).

In the BMAT, ChatGPT performed better in section 1 than in section 2 (*P*=.047), as indicated by higher correct response percentages across all years in section 1, peaking at 66% (17/26) in 2020. Conversely, the model faced difficulties in section 2, especially in 2021, when it achieved only a 5% (1/22) correct response rate. This difference was evident in candidate percentage ranking, with a maximum of 73% (2020) in section 1 showing moderate success, compared to a minimum of 1% (2021) in section 2, emphasizing the model’s struggles in this section.

In the TMUA, ChatGPT demonstrated more consistency in answering questions, achieving a 100% (20/20) response rate in paper 1 (2021) and paper 2 (2019). ChatGPT’s performance was no different in either paper (*P*=.6). Nevertheless, correct response percentages were relatively low, ranging from 11% (2/19) to 22% (4/18) in paper 1 and 11% (2/18) to 20% (4/20) in paper 2. The estimated scores consistently remained low for both papers across all years, with candidate percentage rankings generally below 10%. This suggests that although ChatGPT engaged with the questions, its accuracy in providing correct answers was limited.

In the LNAT, ChatGPT answered all questions in section A of both papers 1 and 2. The correct responses reached 36% (15/42) and 53% (22/42), respectively, indicating a moderately successful performance, particularly in paper 2’s questions. Student performance data for the LNAT were not publicly available.

In the TSA, ChatGPT’s performance in section 1 varied over test years, with the highest correct response percentage in 2019 (22/37, 60%) and the lowest in 2021 (18/43, 42%). The model’s engagement with the questions was relatively high, as the percentage of questions answered ranged from 74% (37/50) to 90% (45/50). The estimated test scores were generally moderate, while candidate percentage ranking fluctuated, with the lowest in 2020 at 9%.

**Table 1:** ChatGPT’s performance on the BioMedical Admissions Test (BMAT), Test of Mathematics for University Admission (TMUA), Law National Aptitude Test (LNAT), and Thinking Skills Assessment (TSA). Performance was measured as percentage of questions that ChatGPT answered correctly and the percentage of questions attempted. The estimated test score and candidate percentage rankings based on ChatGPT’s performance were also derived.

Exam/section			Year		Questions answered, n (%)		Questions correct^a^, n (%)		Test score		Candidate ranking, %
**Biomedical Admissions Test**											
	Section 1 (n=35)	2019		16 (46)		8 (50)		≤4.5		≤62	
	Section 1 (n=32)	2020		26 (82)		17 (66)		≤4.9		≤73	
	Section 1 (n=32)	2021		25 (79)		14 (56)		≤4.2		≤51	
	Section 2 (n=27)	2019		17 (63)		3 (18)		≤2.3		≤7	
	Section 2 (n=27)	2020		20 (75)		9 (45)		≤4.9		≤62	
	Section 2 (n=27)	2021		22 (82)		1 (5)		≤1		≤1	
**Test of Mathematics for University Admission (n=20)**											
	Paper 1	2019		18 (90)		4 (22)		≤2.5		≤18	
	Paper 1	2020		19 (95)		2 (11)		≤1		≤3	
	Paper 1	2021		20 (100)		3 (15)		≤1		≤5	
	Paper 2	2019		20 (100)		4 (20)		≤1		≤8	
	Paper 2	2020		17 (85)		2 (12)		≤1		≤6	
	Paper 2	2021		18 (90)		2 (11)		≤1		≤3	
**Law National Aptitude Test (n=42)**											
	Paper 1, section A	2010		42 (100)		15 (36)		—^b^		—	
	Paper 2, section A	2010		42 (100)		22 (53)		—		—	
**Thinking Skills Assessment (n=50)**											
	Section 1	2019		37 (74)		22 (60)		≤63		≤42	
	Section 1	2020		45 (90)		20 (45)		≤57.5		≤9	
	Section 1	2021		43 (86)		18 (42)		≤57		≤15	

^a^Percentages represent questions correct of questions answered.

^b^Not available.

A similar trend was observed based on test and section when considering the proportion of correct responses to questions of easy to moderate difficulty (BMAT section 1, *P*=.3; BMAT section 2, *P*=.04; TMUA paper 1, *P*<.001; TMUA paper 2, *P*=.003; TSA section 1, *P*=.8; and section A of LNAT papers 1 and 2, *P*>.99) and hard to challenging difficulty (BMAT section 1, *P*=.7; BMAT section 2, *P*<.001; TMUA paper 1, *P*=.007; TMUA paper 2, *P*<.001; TSA section 1, *P*=.3; and section A of LNAT papers 1 and 2, *P*=.2).

## Discussion

### Principal Findings

Our study assessed ChatGPT’s performance on questions derived from various standardized UK admission tests, including the BMAT, TMUA, LNAT, and TSA examinations, to gauge its potential as an innovative tool for education and test preparation in the United Kingdom. We found significant performance variation across different tests and sections. The proportion of correct responses was significantly lower in BMAT section 2 (Scientific Knowledge and Applications) and TMUA papers 1 and 2 (Mathematical Knowledge and Reasoning), while no significant differences were observed in BMAT section 1 (Thinking Skills), TSA section 1 (Problem Solving and Critical Thinking), and section A of LNAT papers 1 and 2 (Comprehension and Reasoning). Hence, ChatGPT performed better in BMAT section 1, TSA section 1, and section A of LNAT papers 1 and 2 but struggled with BMAT section 2 and TMUA papers 1 and 2, exhibiting limited accuracy. Similar trends were observed in ChatGPT’s performance based on question difficulty, consistent for both easy to moderate (quartiles 1 and 2) and hard to challenging (quartiles 3 and 4) questions across tests and sections.

The variations in ChatGPT’s performance across the different tests can be attributed to the distinct skills and aptitudes assessed by each exam. These differences also highlight the model’s strengths and limitations in tackling various subject areas and question formats.

In the BMAT, section 1 assesses thinking skills, which are more general in nature and may align better with the broad training of ChatGPT. This is supported by the stronger performance observed in this section. However, section 2, which focuses on scientific knowledge and applications, proved more challenging for the model. This could be due to the specialized content and context-specific knowledge required, which may not be as thoroughly represented in ChatGPT’s training data.

For the TMUA, the model demonstrated high engagement but limited accuracy in both paper 1 (Mathematical Knowledge and Application) and paper 2 (Mathematical Reasoning). The nature of mathematics questions may require more precise problem-solving skills, which could be challenging for ChatGPT, given its unsupervised learning approach. Additionally, it is possible that the model may not have been exposed to specific mathematical concepts during training or that it lacks the ability to effectively apply them in the context of the TMUA.

In the LNAT, ChatGPT showed moderately successful performance, particularly in paper 2’s reading comprehension questions. This could be attributed to the model’s extensive training in language processing, which allows it to better understand and analyze textual information. However, the lower performance in paper 1, even though papers 1 and 2 both assess the same skills, suggests that the model may have limitations in its ability to adapt to certain question types, arguments, and reasoning tasks.

Finally, in the TSA, the model’s performance varied across test years. The TSA assesses problem-solving and critical thinking skills, which may partially align with the model’s training but still pose challenges due to the diverse range of question types and topics. The fluctuations in performance could indicate that ChatGPT’s success in this test is dependent on the specific content and format of the questions encountered in each year.

As ChatGPT is designed to process and analyze natural language, it is better suited to tasks that involve language understanding and processing, allowing it to identify patterns, make connections between different pieces of information, and generate insights. This makes the AI model particularly effective at tasks that involve complex reasoning and interpretation. However, it is also likely that ChatGPT performs best on shorter, simpler, and clearer questions that are not predicated on background knowledge.

From an education tool perspective, ChatGPT’s performance suggests that it may be more effective in providing support for certain subject areas and test formats in the context of small group learning or virtual tutoring, such as general aptitude, problem-solving and critical thinking, and reading comprehension. However, its limitations in other areas, such as scientific and mathematical knowledge and applications, indicate that it may not yet be a reliable, stand-alone resource for students preparing for these tests. Our findings underscore the importance of integrating ChatGPT into a comprehensive learning strategy without disregarding traditional methods, such as textbooks, lectures, and tutoring sessions with subject matter experts. Moreover, educators and researchers should continue to explore ways to optimize ChatGPT’s performance in areas where it currently struggles, potentially by refining its training data or incorporating specialized knowledge and algorithms.

From an ethical standpoint, the potential misuse of AI tools like ChatGPT for cheating or gaining unfair advantages in admission tests is a significant concern. In our study, we focused on evaluating ChatGPT as an educational tool for test preparation, rather than promoting its use during actual exams. Our findings indicate that given its limitations and varying performance across different subject areas and test formats, it is currently not feasible for ChatGPT to provide a substantial unfair advantage to test-takers. However, as AI models like ChatGPT continue to improve through better training data and more advanced algorithms, increasingly accurate language models and the ability to generate more contextually relevant responses are becoming the norm. This progress ushers in a new frontier of ethical considerations for their use in educational settings.

We believe that AI tools can be valuable for education if used ethically and responsibly, aiming to enhance learning experiences and test preparation. In the future, it will be crucial for stakeholders, including educational institutions, test administrators, and AI developers, to collaboratively establish guidelines and preventive measures to ensure ethical and responsible AI use in education. Potential strategies may involve developing sophisticated methods for detecting AI-generated content during exams, incorporating secure proctoring systems, and providing comprehensive education on the ethical use of AI tools for students, educators, and test-takers. By proactively addressing these ethical concerns, we can harness the potential benefits of AI tools like ChatGPT while mitigating the risks associated with their misuse.

### Limitations

There are several limitations to our study. First, we only evaluated ChatGPT’s performance on a limited number of standardized admission tests in the United Kingdom, which may not be representative of all tests used in other countries or academic programs. Second, the study is constrained by the fact that ChatGPT was trained on a corpus of data produced on or before 2021, limiting its exposure to information beyond that time frame. This could impact its ability to handle contemporary problems or novel scenarios that arise after 2021. Third, as ChatGPT is designed to process and analyze natural language, it may not be as effective in handling certain types of mathematically intensive questions that require advanced knowledge or abstract concepts. Fourth, our study evaluated only ChatGPT’s performance and did not compare it to other AI models or to human performance. Lastly, ChatGPT is continually updated, and the version used in our study may not represent the most recent iteration at the time of publication. Despite these limitations, our study provides valuable insights into the strengths and limitations of ChatGPT in the context of standardized admission tests in the United Kingdom. Further research is needed to explore its potential in other educational contexts and to further address its limitations as an innovative tool for education and test preparation.

### Conclusions

Our study evaluated ChatGPT’s performance on various standardized admission tests in the United Kingdom and found that the model exhibited variations in performance across different test types and sections. While ChatGPT has potential as a supplemental educational tool, its limitations and capabilities must be carefully considered in the context of specific subject areas and test formats. The advent of ChatGPT has sparked concerns about its impact on exam assessment processes, the educational system, and university programs. Future research should address the limitations identified in our study to enhance ChatGPT’s effectiveness as an educational tool in broader educational contexts.

## Abbreviations

BMAT: BioMedical Admissions Test
LNAT: Law National Aptitude Test
TMUA: Test of Mathematics for University Admission
TSA: Thinking Skills Assessment
USMLE: United States Medical Licensing Examination

## Data Availability

The data sets generated during and/or analyzed during this study are available from the corresponding author on reasonable request.
